# The Role of Incentive Respiratory Techniques in Enhanced Recovery After Lung Cancer Resection: A Propensity Score-Matched Study

**DOI:** 10.3390/jcm14010100

**Published:** 2024-12-27

**Authors:** Monica Casiraghi, Riccardo Orlandi, Luca Bertolaccini, Antonio Mazzella, Lara Girelli, Cristina Diotti, Giovanni Caffarena, Silvia Zanardi, Federica Baggi, Francesco Petrella, Patrick Maisonneuve, Lorenzo Spaggiari

**Affiliations:** 1 Department of Thoracic Surgery, European Institute of Oncology (IEO) IRCCS, 20141 Milan, Italy; luca.bertolaccini@ieo.it (L.B.); antonio.mazzella@ieo.it (A.M.); lara.girelli@ieo.it (L.G.); cristina.diotti@ieo.it (C.D.); giovanni.caffarena@ieo.it (G.C.); francesco.petrella@ieo.it (F.P.); lorenzo.spaggiari@ieo.it (L.S.); 2 Department of Oncology and Hemato-Oncology, University of Milan, 20122 Milan, Italy; 3 Department of Thoracic Surgery, University of Milan, 20122 Milan, Italy; riccardo.orlandi@unimi.it; 4 Division of Physiotherapy, European Institute of Oncology (IEO) IRCCS, 20141 Milan, Italy; silvia.zanardi@ieo.it (S.Z.); federica.baggi@ieo.it (F.B.); 5 Division of Epidemiology and Biostatistics, European Institute of Oncology (IEO) IRCCS, 20141 Milan, Italy; patrick.maisonneuve@ieo.it

**Keywords:** lung cancer, physiotherapy, ERAS, lung resection, early ambulation, incentive respiratory techniques

## Abstract

**Background:** Postoperative physiotherapy is a cornerstone of Enhanced Recovery After Surgery (ERAS) programs, especially following lung resection. Despite its importance, the literature lacks clear recommendations and guidelines, particularly regarding the role of Incentive Respiratory Techniques (IT). This study aims to determine whether IT offers additional benefits over early ambulation in patients undergoing lung resection for primary lung cancer. **Methods:** We conducted a retrospective case–control study at the European Institute of Oncology (IEO) involving patients who underwent lung resection from February 2019 to June 2022. Patients were divided into two cohorts: early ambulation (control group) and early ambulation with IT (IT group). The primary endpoint was the rate of postoperative pulmonary complications. Secondary endpoints included length of hospital stay and time to chest drain removal. A propensity score-matched analysis was performed based on age, sex, and BMI. Data were compared using Chi-squared and Student’s *t*-tests as appropriate. **Results:** A total of 304 patients were included, with 153 in the intervention group and 151 in the control group. After propensity-score matching, 52 patients from each cohort were compared. No significant differences were found between the groups regarding postoperative oxygen requirement, fever, atelectasis, residual pleural space, need for bronchoscopy toilette, and re-hospitalization rate. IT group showed trends toward shorter hospital stays and lower time to chest drain removal, though without reaching statistical significance. **Conclusions:** IT did not significantly improve postoperative outcomes compared to early ambulation in patients undergoing lung resection for primary lung cancer. More extensive, prospective, randomized trials are needed to confirm these findings.

## 1. Introduction

Despite ongoing improvements in surgical outcomes, ensured by technological development and technical refinement, postoperative complications still burden the course after surgery [1]. Enhanced Recovery After Surgery (ERAS) protocols are designed to optimize perioperative care, minimizing complications and expediting recovery [2]. Briefly, ERAS programs aim at maximizing preoperative patients’ condition and resuming the highest achievable functional level as soon as possible after surgery, by proposing a bundle of multimodal interventions to be adopted in the perioperative course [3]. Postoperative physiotherapy is a critical component of these protocols, particularly following lung resection [4], allowing re-inflation of collapsed alveoli, an increase of lung compliance, as well as a decrease of perfusion-ventilation inequities. Indeed, lung surgery is still affected by a relatively high rate of postoperative complications compared to other surgeries, mainly due to altered breathing mechanics, secretion retention, and atelectasis, ultimately leading to a reduction in respiratory function and pulmonary complications [5,6]. Volume reduction, lung compression, prolonged lateral and supine positioning, pain, respiratory muscle impairment, barotrauma, and volume trauma related to mechanical ventilation compete in the genesis of complications [7]. Proactive postoperative physiotherapy could virtually decrease the incidence of postoperative pulmonary complications [8]. However, more definitive guidelines must be provided regarding the best physiotherapeutic practices in this context. Incentive respiratory techniques (IT) and positive expiratory pressure (PEP) are often prescribed postoperatively to improve pulmonary function and reduce complications post-surgery [9] by promoting alveolar expansion and fostering mobilization of secretions; therefore, it is widely spread in routine practice, because it is usually safe, well tolerated by the patients, and with high compliance, unless the patients had previous oral surgery which could make difficult or impossible to perform the exercise. Nonetheless, its real efficacy remains unclear. Previous studies have shown conflicting results concerning the routinary use of IT in the postoperative course [10,11,12,13,14,15,16,17,18,19], some demonstrating no clear benefits for IT, though with low-quality evidence, others proving substantial enhancement of postoperative outcomes, though with limited sample size or unclear study design. This study aims to investigate whether the addition of IT to early ambulation offers substantial benefits in the postoperative recovery of lung cancer patients undergoing surgical resection.

## 2. Materials and Methods

### 2.1. Study Design and Population

A retrospective case–control study was conducted at a tertiary referral center for lung cancer, the European Institute of Oncology (IEO) in Milan, Italy. Patients undergoing lung resection such as sublobar resection, lobectomy, or pneumonectomy, for primary lung cancer between February 2019 and June 2022 were included in the study; there were no restrictions either on the choice of surgical approach or on the extent of the planned lung resection. Patients with severe preoperative impaired pulmonary function (“borderline” operable), those unable to ambulate or to understand and follow perioperative instructions, those undergoing surgery for other-than-primary lung cancers or for other-than-pulmonary resections, as well as those with incomplete follow-up were excluded. The follow-up was defined as complete if patients had accomplished a 1-month chest X-ray and outpatient visit.

For all patients, the preoperative staging was conducted within the five weeks prior to surgery, and included a total body computed-tomography (CT) scan and a fluorodeoxyglucose (FDG)-positron emission tomography (PET) scan. Whenever appropriate, any suspected involvement of mediastinal lymph nodes was confirmed using endobronchial ultrasound-guided transbronchial needle aspiration (EBUS-TBNA) or mediastinoscopy. Functional assessment included cardiological examination, pulmonary function test, and blood gas analysis. When recommended, lung perfusion scan and cardiopulmonary exercise testing were performed. Patient’s operability was decided by dedicated anesthesiologists, whereas resectability, extent of resection, surgical approach, and whole treatment plan were discussed in multidisciplinary meetings, comprising thoracic surgeons, radiologists, pathologists, radiotherapists, and oncologists.

Open surgery was performed through lateral totally muscle-sparing thoracotomy in IV or V intercostal space. Minimally invasive surgery was performed through either a robotic or thoracoscopic approach. The former consisted of three-port incisions and a 3 cm utility incision without rib spreading; the latter consisted of a 3 cm utility incision without rib spreading and an additional port for the camera.

After surgery, patients were admitted to the Intensive Care Unit for monitoring for the first 24 h if required; otherwise, they returned immediately back to the ward. Usually, we followed the ERAS program with early mobilization (within 12 h), and respiratory physiotherapy.

Patients were retrospectively divided into two cohorts based on their postoperative physiotherapy protocol: those who received early ambulation (control group) and those who received early ambulation with IT (IT group). Patients in the control group underwent routine physiotherapy care based on early ambulation on the first postoperative day (at least 12 h after the surgery), consisting of active and assisted walking (keeping a diary for goals, gradually increasing in terms of frequency and quantity of walking over the days) for at least 5 min each hour for at least 8 h per day, in addition to daily visits by physiotherapist teaching deep breathing (the patient was instructed to maintain a sustained maximal inspiration for 7 s before exhalation), coughing, and chest wall reinforcement exercises, which the patients were encouraged to perform independently after discharge until the outpatient visit at 30 days from surgery. Patients in the IT group received PEP in addition to the above-mentioned routine physiotherapy; the PEP exercise was performed by blowing into a tube submerged 10 cm in a bottle of water, with 3 sets of 10 breaths approximately every hour. At the end of each set, an active cycle of breathing exercise was performed: controlled breathing—slow and prolonged inspiration—inspiratory pause with an open glottis—forced and prolonged expiration. Again, the patients were promoted to perform the exercises daily after discharge, according to the in-hospital schedule. Unlike the control group, the IT group did not keep a walking diary. Patients were assigned to undergo IT or early ambulation based on the year in which they underwent surgery, on the physiotherapist available at the ward at that moment, on their personal preference. Patients underwent previous oral surgery, which could make it difficult or impossible to perform the exercise, were excluded from the study.

Postoperative analgesia in both cohorts was managed in collaboration with anesthesiologists, utilizing a combination of epidural analgesia (when technically feasible and not contraindicated), patient-controlled analgesia, and oral and parenteral adjuncts as needed to enhance pulmonary and physical therapy.

Postoperative thromboprophylaxis was managed through subcutaneous low-molecular-weight heparin, together with compression stockings and early ambulation.

A chest drain was removed if effusion output was equal or inferior to 300 mL/24 h, without air leak for at least 12 h. Patients received daily chest X-rays and blood tests until discharge. Then, patients were followed up with an outpatient visit 1 month after surgery, with chest X-ray and blood tests.

### 2.2. Data Collection and Variables

Data were retrieved from patient medical records, demographic characteristics, smoking habits, previous surgeries, extent of resection, access type, and postoperative course.

The primary endpoint was the rate of postoperative pulmonary complications occurring within 30 days after surgery, including oxygen requirement, fever, atelectasis, residual pleural space, need for bronchoscopy toilette, and re-hospitalization rate. The Ottawa Thoracic Morbidity and Mortality System was used to classify and to grade surgical complications [20].

Secondary endpoints were the length of hospital stay and the time to chest drain removal.

The study was conducted in accordance with the Declaration of Helsinki. No authorization from our ethics committee was required because the applied device was already approved in real life for the evaluation of vital parameters, both in basal conditions and under stress. Written informed consent was obtained from all patients at the time of surgery. All data underlying this article are available in the article.

### 2.3. Statistical Analysis

To account for potential confounders, a propensity score-matched analysis was performed based on age, sex, and BMI [21]. Specifically, patients within the IT cohort were coupled to those of the control group with similar estimated propensity scores, considering age bracket (<60, 60–69, 70+), gender, and BMI range (18.5–24.9, 25.0–29.9, 30+). Descriptive data were reported as numbers and percentages for categorical variables and medians and ranges for continuous variables. Comparative analyses were conducted using Chi-squared tests for categorical data and Student’s *t*-tests or Mann–Whitney U test for continuous data, as appropriate. Analyses were performed using SAS software v. 9.4 (SAS Institute, Cary, NC, USA). A *p*-value of less than 0.05 was considered statistically significant.

## 3. Results

### 3.1. Patient Characteristics

A total of 304 patients fulfilled the inclusion criteria and were enrolled in the study: 153 in the IT group and 151 in the control group. All patients in the IT group well tolerated the performance required, with 97% compliance; only three patients were not able to perform the exercise, due to previous laryngectomy or oral surgery which made it difficult or impossible to perform the exercise. These three patients were excluded from the propensity-score matching. Demographic characteristics are reported in Table 1. After propensity-score matching, 52 patients from each cohort were compared, ensuring balanced baseline characteristics (48.1% female, median age 65, median BMI 25.4). The majority were aged between 60 and 69 years old (*n* = 42; 40.4%), male (*n* = 54; 51.9%), with normal weight (*n* = 50, 48.1%). Most patients were former smokers (*n* = 55; 52.9%) and without previous lung surgery (*n* = 93; 89.4%). Only a minority received neoadjuvant therapy (*n* = 11; 10.6%). The majority of patients underwent pulmonary lobectomy (*n* = 56; 53.8%) through either open thoracotomy (*n* = 52; 50%) or a minimally invasive approach (*n* = 52; 50%), whereas the remainder underwent sub-lobar resection (*n* = 34; 32.7%), bi-lobectomy (*n* = 4; 3.8%), or pneumonectomy (*n* = 10; 9,6%). No significant differences concerning perioperative variables between the two cohorts were recorded (Table 1).

### 3.2. Primary and Secondary Outcomes

Clinical outcomes are reported in Table 2. Within the IT group, nine patients (17.3%) required oxygen, nine patients (17.3%) had a fever, one (1.9%) developed atelectasis, forty-one (78.8%) had residual pleural space, five (9.6%) needed bronchoscopy toilette, and three (5.8%) experienced re-hospitalization; on the other hand, within the control group, six patients (11.5%) required oxygen, four (7.7%) had a fever, one (1.9%) developed atelectasis, thirty-six (69.2%) had residual pleural space, five (9.6%) needed bronchoscopy toilette, and two (3.8%) experienced re-hospitalization. Patients in the IT cohort had a median hospital stay of 4.5 days and a median time to chest drain removal of 3.0 days, whereas those in the control cohort had a median hospital stay of 5 days and a median time to chest drain removal of 4.0 days. No statistically significant differences were observed between the two groups in terms of postoperative oxygen requirement (17.3% vs. 11.5%, *p* = 0.40), fever (17.3% vs. 7.7%, *p* = 0.14), atelectasis (1.9% vs. 1.9%, *p* = 1.00), residual pleural space (78.8% vs. 69.2%, *p* = 0.26), need for bronchoscopy toilette (9.6% vs. 9.6%, *p* = 1.00), and re-hospitalization rate (5.8% vs. 3.8%, *p* = 0.65). Again, secondary outcomes failed to reach statistically significant differences, though potential clinically significant differences have been recorded: indeed, the IT group showed a non-significant trend towards shorter hospital stays (4.5 days vs. 5 days, *p* = 0.82) and quicker chest drain removal (3 days vs. 4 days, *p* = 0.49), which are noteworthy to be highlighted, due to their clinical impact on nowadays routinary practice and which may become statistically significant on larger sample size.

## 4. Discussion

IT is still widely adopted throughout several Thoracic Surgery Units after lung resection despite its effectiveness being highly debated. Indeed, though resulting from limited and mixed evidence, IT is thought to promote alveolar expansion and to foster mobilization of secretions, leading to faster pulmonary function recovery and reduced postoperative complications, by maximizing motivation and accuracy of breathing.

So far, very limited and discordant data are available in the literature about the use and effectiveness of IT on pulmonary postoperative outcomes, mainly even based on cardiac or abdominal surgery [22,23,24,25] rather than on pulmonary resection. In detail, Freitas and colleagues [22] concluded that IT does not add any benefit over standard physical therapy for preventing postoperative complications or reducing the length of hospital stay in patients undergoing coronary artery bypass grafting; Pasquina and colleagues [23] showed that routine use of respiratory physiotherapy after abdominal surgery does not seem to be justified. On the other hand, Hall and colleagues [24] proved that IT is the most efficient prophylaxis method against respiratory complications in high-risk patients undergoing abdominal surgery; similar results were given by Thomas and McIntosh [25]. Overend and colleagues [26], in 2001, systematically reviewed the evidence published in the literature examining the use of IT for the prevention of postoperative pulmonary complications. Still, only 11 out of 46 analyzed studies were shown to state an adequate conclusion, amongst which 10 did not support the use of IT for decreasing the incidence of pulmonary complications following cardiac or upper abdominal surgery. A prospective randomized controlled trial published in 2013 by Malik and colleagues [9] and conducted in adults undergoing lung resection showed that the addition of IT to routine postoperative physiotherapy did not reduce the incidence of postoperative pulmonary complications compared with physiotherapy alone. Even our study found no significant differences in postoperative complications such as oxygen requirement, fever, atelectasis, residual pleural space, need for bronchoscopy toilette, and re-hospitalization rate between the two groups, confirming the literature findings [9,26], and questioning the use of IT in preventing postoperative pulmonary complications in thoracic surgery patients. The theoretical benefits of IT include improved lung expansion and prevention of atelectasis by encouraging sustained maximal inspiration [27]. However, the practical efficacy of this intervention on postoperative complications, in particular pulmonary, has been debated. Some studies [28,29] found that while incentive spirometry can enhance lung function parameters, these improvements do not necessarily translate into clinically significant benefits in postoperative outcomes: in particular, Barbalho-Moulim and colleagues [28] showed that respiratory training could enhance muscle strength, though without improving lung volumes or diaphragmatic excursion; whereas Carvalho and colleagues [29] found that though offering some potential advantages over standard postoperative physiotherapy, incentive spirometry does not modify the outcomes. The missed translation from improved function parameters to clinically significant outcomes could be related to different factors. First of all, the proper use of incentive spirometry requires patient compliance and correct technique, and it could vary widely among individuals, highlighting the challenges in ensuring consistent and effective use of IT among patients [30,31,32]. According to our experience, proper preoperative and perioperative counseling and training in using IT are fundamental to gaining fair adherence of patients to scheduled therapy: the main difficulty reported by our patients was the intensive program scheduled for post-discharge training; nevertheless, the reported adherence was satisfactory. Furthermore, the baseline respiratory function of patients could influence the effectiveness of IT since patients with better preoperative pulmonary function might benefit less from additional respiratory exercises postoperatively [33,34].

Therefore, the results of this study suggest that the addition of IT to early ambulation does not significantly impact postoperative outcomes in patients undergoing lung resection for primary lung cancer. Despite trends towards shorter hospital stays and quicker chest drain removal in the IT group, these differences did not reach statistical significance, overlapping with other studies [22,35], which found similar non-significant trends when comparing IT with other physiotherapeutic interventions, though in the different setting of postoperative course after coronary artery bypass grafting. Based on these results, we changed our postoperative physiotherapy treatment, shifting toward early ambulation, considering IT not improving the postoperative outcome of the patients.

This aligns with some existing literature [9,36,37,38,39,40], highlighting the need for further investigation into the role of IT in ERAS protocols, which usually include multiple interventions aimed at optimizing recovery, which may dilute the apparent impact of any single component, such as IT [41,42].

This study has some limitations. The retrospective design may have introduced inherent biases. Furthermore, the single-center nature of the study might limit the generalizability of the findings to other settings or populations. The random assignment of a patient to undergo IT or early ambulation, based on the physiotherapist available at the ward at that moment and their personal preference, could be a bias; however, we realize that a randomized study would have granted a higher statistical power, but our retrospective matching represents a “real-life” experience, or a daily clinical routine practice, which is more reproducible in other centers. Data concerning comorbidities, especially chronic obstructive pulmonary disease and relative treatments, were largely missing, thus potentially limiting the generalizability of the results within this specific cohort of patients. Additionally, while propensity score matching helps control for some confounders, unmeasured variables could still affect the results. Then, the extent of surgical resections resulted to be slightly different between the two matched cohorts, though without statistical significance, thus potentially introducing a selection bias. Finally, the sample size, particularly after matching, may not have been large enough to detect subtle differences between groups. Future research should focus on larger, prospective, randomized controlled trials to confirm these findings. Studies should also explore patient-specific factors, such as baseline respiratory function and adherence to postoperative physiotherapy protocols, to understand better who might benefit most from IT. Additionally, integrating objective measures of lung function and patient-reported outcomes could provide a more comprehensive assessment of the efficacy of IT.

## 5. Conclusions

In this retrospective case–control study, IT did not significantly improve postoperative outcomes in lung resection patients compared to early ambulation. Although slight trends towards shorter hospital stays and quicker chest drain removal were noted, these findings were not statistically significant. Future research with a larger sample size and randomized controlled design is necessary to confirm these results and potentially refine postoperative physiotherapy guidelines in ERAS protocols.

## Figures and Tables

**Table 1:** Comparison between IT and early ambulation groups, before and after propensity-score matching: baseline and perioperative characteristics.

	Unmatched				Matched		
	All	Incentive Respiratory Technique (IT Group)	Early Ambulation (Control Group)	*p*-Value	Incentive Respiratory Technique (IT Group)	Early Ambulation (Control Group)	*p*-Value
Total	304	153	151		52	52	
Age							
Median [range]	65.5 [25–85]	64.0 [33–85]	67.0 [25–82]	0.01	65 [34–84]	65 [32–79]	0.88
<60	92 (30.3)	56 (36.6)	36 (23.8)		15 (28.8)	15 (28.8)	
60–69	106 (34.9)	52 (34.0)	54 (35.8)		21 (40.4)	21 (40.4)	
70+	106 (34.9)	45 (29.4)	61 (40.4)	0.03	16 (30.8)	16 (30.8)	0.76
Sex							
Women	170 (55.9)	118 (77.1)	52 (34.4)		25 (48.1)	25 (48.1)	
Men	134 (44.1)	35 (22.9)	99 (65.6)	<0.0001	27 (51.9)	27 (51.9)	0.92
BMI							
Median [range]	25.2 [16.4–45.7]	22.9 [16.4–45.7]	28.1 [20.0–37.3]	<0.0001	25.1 [17.7–37.8]	25.7 [20.0–34.2]	0.65
Normal weight	144 (47.4)	117 (76.5)	27 (17.9)		25 (48.1)	25 (48.1)	
Overweight	109 (35.9)	25 (16.3)	84 (55.6)		20 (38.5)	20 (38.5)	
Obese	51 (16.8)	11 (7.2)	40 (26.5)	<0.0001	7 (13.5)	7 (13.5)	0.78
Smoking							
Never	88 (28.9)	56 (36.6)	32 (21.2)		17 (32.7)	9 (17.3)	
Former	149 (49.0)	62 (40.5)	87 (57.6)		23 (44.2)	32 (61.5)	
Current	67 (22.0)	35 (22.9)	32 (21.2)	0.004	12 (23.1)	11 (21.2)	0.14
Previous lung surgery							
No	265 (87.2)	134 (87.6)	131 (86.8)		46 (88.5)	47 (90.4)	
Yes	39 (12.8)	19 (12.4)	20 (13.2)	0.83	6 (11.5)	5 (9.6)	0.75
Neoadjuvant therapy							
None	266 (87.5)	132 (86.3)	134 (88.7)		46 (88.5)	47 (90.4)	
CT	36 (11.8)	19 (12.4)	17 (11.3)		4 (7.7)	5 (9.6)	
RT	1 (0.3)	1 (0.7)	0 (0.0)		1 (1.9)	0 (0.0)	
CT-RT	1 (0.3)	1 (0.7)	0 (0.0)	0.55	1 (1.9)	0 (0.0)	0.55
Type of surgery							
Sublobar	127 (41.8)	74 (48.4)	53 (35.1)		22 (42.3)	12 (23.1)	
Lobectomy	154 (50.7)	70 (45.8)	84 (55.6)		25 (48.1)	31 (59.6)	
Bilobectomy	6 (2.0)	2 (1.3)	4 (2.6)		1 (1.9)	3 (5.8)	
Pneumonectomy	17 (5.6)	7 (4.6)	10 (6.6)	0.12	4 (7.7)	6 (11.5)	0.17
Access							
Thoracotomy	154 (50.7)	77 (50.3)	77 (51.0)		26 (50.0)	26 (50.0)	
VATS	68 (22.4)	41 (26.8)	27 (17.9)		13 (25.0)	8 (15.4)	
RATS	82 (27.0)	35 (22.9)	47 (31.1)	0.10	13 (25.0)	18 (34.6)	0.37

**Table 2:** Comparison between IT and early ambulation groups, before and after propensity-score matching: postoperative outcomes.

	Unmatched				Matched		
	All	Incentive Respiratory Technique (IT Group)	Early Ambulation (Control Group)	*p*-Value	Incentive Respiratory Technique (IT Group)	Early Ambulation (Control Group)	*p*-Value
Total	304	153	151		52	52	
Oxygen requirement							
No	254 (83.6)	133 (86.9)	121 (80.1)		43 (82.7)	46 (88.5)	
Yes	50 (16.4)	20 (13.1)	30 (19.9)	0.11	9 (17.3)	6 (11.5)	0.40
Fever							
No	272 (89.5)	137 (89.5)	135 (89.4)		43 (82.7)	48 (92.3)	
Yes	32 (10.5)	16 (10.5)	16 (10.6)	0.97	9 (17.3)	4 (7.7)	0.14
Atelectasis							
No	295 (97.0)	150 (98.0)	145 (96.0)		51 (98.1)	51 (98.1)	
Yes	9 (3.0)	3 (2.0)	6 (4.0)	0.30	1 (1.9)	1 (1.9)	1.00
Residual pleural space							
No	75 (24.7)	27 (17.6)	48 (31.8)		11 (21.2)	16 (30.8)	
Yes	229 (75.3)	126 (82.4)	103 (68.2)	0.004	41 (78.8)	36 (69.2)	0.26
Bronchoscopy toilette							
No	278 (91.4)	145 (94.8)	133 (88.1)		47 (90.4)	47 (90.4)	
Yes	26 (8.6)	8 (5.2)	18 (11.9)	0.04	5 (9.6)	5 (9.6)	1.00
Re-hospitalization							
No	290 (95.4)	144 (94.1)	146 (96.7)		49 (94.2)	50 (96.2)	
Yes	14 (4.6)	9 (5.9)	5 (3.3)	0.28	3 (5.8)	2 (3.8)	0.65
Hospital stay							
Median [range]	5.0 [2–19]	4.0 [2–18]	5.0 [2–19]	0.17	4.5 [2–18]	5.0 [2–12]	0.82
Drainage							
Median [range]	4.0 [1–18]	3.0 [1–16]	4.0 [1–18]	0.03	3.0 [1–16]	4.0 [1–12]	0.49

## Data Availability

The original contributions presented in this study are included in the article.
