# Correction: Casiraghi et al. The Role of Incentive Respiratory Techniques in Enhanced Recovery After Lung Cancer Resection: A Propensity Score-Matched Study. *J. Clin. Med.* 2025, *14*, 100

**DOI:** 10.3390/jcm15020529

**Published:** 2026-01-09

**Authors:** Monica Casiraghi, Riccardo Orlandi, Luca Bertolaccini, Antonio Mazzella, Lara Girelli, Cristina Diotti, Giovanni Caffarena, Silvia Zanardi, Federica Baggi, Francesco Petrella, Patrick Maisonneuve, Lorenzo Spaggiari

**Affiliations:** 1Department of Thoracic Surgery, European Institute of Oncology (IEO) IRCCS, 20141 Milan, Italy; luca.bertolaccini@ieo.it (L.B.); antonio.mazzella@ieo.it (A.M.); lara.girelli@ieo.it (L.G.); cristina.diotti@ieo.it (C.D.); giovanni.caffarena@ieo.it (G.C.); francesco.petrella@ieo.it (F.P.); lorenzo.spaggiari@ieo.it (L.S.); 2Department of Oncology and Hemato-Oncology, University of Milan, 20122 Milan, Italy; 3Department of Thoracic Surgery, University of Milan, 20122 Milan, Italy; riccardo.orlandi@unimi.it; 4Division of Physiotherapy, European Institute of Oncology (IEO) IRCCS, 20141 Milan, Italy; silvia.zanardi@ieo.it (S.Z.); federica.baggi@ieo.it (F.B.); 5Division of Epidemiology and Biostatistics, European Institute of Oncology (IEO) IRCCS, 20141 Milan, Italy; patrick.maisonneuve@ieo.it

## Text Correction

There was an error in the original publication [1]. The method applied in the intervention group is better defined as an incentive respiratory technique rather than as incentive spirometry, since a spirometer tool was not applied and defining it as incentive spirometry could be misleading. 

So, the name of the intervention group was changed from the incentive spirometry group to the incentive respiratory technique (IT) group. In the text, except for the following sentences in the Discussion section, every time there is “incentive spirometry” has to be changed to “incentive respiratory technique”, and the acronym IS has to be changed to IT.

“However, the practical efficacy of this intervention on postoperative complications, in particular pulmonary, has been debated. Some studies [28,29] found that while incentive spirometry can enhance lung function parameters, these improvements do not necessarily translate into clinically significant benefits in postoperative outcomes: in particular, Barbalho-Moulim and colleagues [28] showed that respiratory training could enhance muscle strength, though without improving lung volumes or diaphragmatic excursion; whereas Carvalho and colleagues [29] found that though offering some potential advantages over standard postoperative physiotherapy, incentive spirometry does not modify the outcomes. The missed translation from improved function parameters to clinically significant outcomes could be related to different factors. First of all, the proper use of incentive spirometry requires patient compliance and correct technique”.

The correction of the name of the intervention group has been applied to the Title, Abstract, Introduction, Materials and Methods, Results, Tables, Discussion, and Conclusions. As well, the word “alone” was erased from the description of the control group all over the manuscript. The modified “Methods” paragraph is reported below, whereas the other paragraphs contain only minor changes as you can see below:

In the Introduction, we modified the phrase adding the positive expiratory pressure (PEP) and erasing part of the phrase as shown below.

“Incentive spirometry (IS), a common respiratory therapy technique based on deep breathing through a device that provides visual feedback maximizing motivation and accuracy of breathing, is purported to improve pulmonary function and reduce complications post-surgery [9]” changed to

“Incentive respiratory techniques (IT) and positive expiratory pressure (PEP) are often prescribed postoperatively to improve pulmonary function and reduce complications post-surgery [9]”.

In the Abstract section, the period of the study was corrected from “June 2020 to June 2022” to “February 2019 to June 2022”. In the Materials and Methods section, the period of the study was corrected from “between June 2020 and June 2022” to “between February 2019 and June 2022”.

In the Materials and Methods section, we added the description of the incentive respiratory technique used and erased the other technique, as shown in the paragraphs below. To make it clearer the correction we reported below the final paragraph.

“Patients were retrospectively divided into two cohorts based on their postoperative physiotherapy protocol: those who received early ambulation alone (control group) and those who received early ambulation with IS (IS group). Patients in the control group underwent routine physiotherapy care based on early ambulation on the first postoperative day (at least 12 h after the surgery), consisting of active and assisted walking for at least 5 min each hour for at least 8 h per day, in addition to daily visits by physiotherapist teaching deep breathing, coughing, and chest wall reinforcement exercises, which the patients were encouraged to perform independently after discharge until the outpatient visit at 30 days from surgery. Patients in the IS group received IS in addition to the above-mentioned routine physiotherapy; an incentive spirometer (AirLife^TM^, Walker, MI, USA) was provided on the first postoperative day, and the patient was encouraged to use it immediately, at least 10 min every 2 h on seated position while awake, training on slow deep breathing and forced expiratory breathing, gradually increasing the device’s negative pressure threshold, after being properly instructed on its use. The patient was instructed to maintain a sustained maximal inspiration for 5 s before exhalation. Again, the patients were promoted to perform the exercises daily after discharge, according to the in-hospital schedule. Patients were randomly assigned to undergo IS or early ambulation alone based on the physiotherapist available at the ward at that moment, on their personal preference, and on the previous patient’s history of oral surgery, which could make it difficult or impossible to perform the exercise.” changed to

“Patients were retrospectively divided into two cohorts based on their postoperative physiotherapy protocol: those who received early ambulation (control group) and those who received early ambulation with IT (IT group). Patients in the control group underwent routine physiotherapy care based on early ambulation on the first postoperative day (at least 12 h after the surgery), consisting of active and assisted walking (keeping a diary for goals, gradually increasing in terms of frequency and quantity of walking over the days) for at least 5 min each hour for at least 8 h per day, in addition to daily visits by physiotherapist teaching deep breathing (the patient was instructed to maintain a sustained maximal inspiration for 7 s before exhalation), coughing, and chest wall reinforcement exercises, which the patients were encouraged to perform independently after discharge until the outpatient visit at 30 days from surgery. Patients in the IT group received PEP in addition to the above-mentioned routine physiotherapy; the PEP exercise was performed by blowing into a tube submerged 10 cm in a bottle of water, with 3 sets of 10 breaths approximately every hour. At the end of each set, an active cycle of breathing exercise was performed: controlled breathing—slow and prolonged inspiration—inspiratory pause with an open glottis—forced and prolonged expiration. Again, the patients were promoted to perform the exercises daily after discharge, according to the in-hospital schedule. Unlike the control group, the IT group did not keep a walking diary. Patients were assigned to undergo IT or early ambulation based on the year in which they underwent surgery, on the physiotherapist available at the ward at that moment, on their personal preference. Patients underwent previous oral surgery, which could make it difficult or impossible to perform the exercise, were excluded from the study.”

In the Institutional Board Statement section, “Data was retrieved from the study UID 3516/03122021.” was added.

## Table Legend

In the original publication, there was a mistake in the legend for Tables 1 and 2. Namely, “IS” was changed to “IT”. The correct legend appears below.

“**Table 1.** Comparison between IT and early ambulation groups, before and after propensity-score matching: baseline and perioperative characteristics.”

“**Table 2.** Comparison between IT and early ambulation groups, before and after propensity-score matching: postoperative outcomes.”

## Error in Table

In the original publication, there was a mistake in the headers of Tables 1 and 2 as published. Namely, “Incentive Spirometry” was changed to “Incentive Respiratory Technique (IT Group)”, “Early Ambulation” was changed to “Early Ambulation (Control Group)”. The corrected Tables 1 and 2 appear below.

**Unmatched****Matched****All****Incentive Respiratory Technique** **(IT Group)****Early Ambulation** **(Control Group)*****p*-Value****Incentive Respiratory Technique** **(IT Group)****Early Ambulation** **(Control Group)*****p*-Value**

## References

The following references need to be replaced to ensure a correct match with the reference citation in the main text.

Reference [12] has to be replaced by the following: Larsen, T.; Chuang, K.; Patel, S.; Betancourt, J. Things We Do for No Reason™: Routine use of postoperative incentive spirometry to reduce postoperative pulmonary complications. *J. Hosp. Med.*
**2022**, *17*, 1010–1013. 

Reference [27] has to be replaced by the following: Orman, J.; Westerdahl, E. Chest physiotherapy with positive expiratory pressure breathing after abdominal and thoracic surgery: a systematic review. *Acta Anaesthesiol. Scand.*
**2010**, *54*, 261–267. 

Reference [34] has to be replaced by the following: Baneton, S.; Dauvergne, J.E.; Gouillet, C.; Cartron, E.; Volteau, C.; Nicolet, J.; Corne, F.; Rozec, B. Effect of Active Physiotherapy With Positive Airway Pressure on Pulmonary Atelectasis After Cardiac Surgery: A Randomized Controlled Study. *J. Cardiothorac. Vasc. Anesth.*
**2023**, *37*, 1668–1676. 

Reference [38] has to be replaced with the following: Iverson, L.I.; Ecker, R.R.; Fox, H.E.; May, I.A. A comparative study of IPPB, the incentive spirometer, and blow bottles: the prevention of atelectasis following cardiac surgery. *Ann. Thorac. Surg.*
**1978**, *25*, 197–200. 

Reference [39] has to be replaced with the following: Hanada, M.; Kanetaka, K.; Hidaka, S.; Taniguchi, K.; Oikawa, M.; Sato, S.; Eguchi, S.; Kozu, R. Effect of early mobilization on postoperative pulmonary complications in patients undergoing video-assisted thoracoscopic surgery on the esophagus. *Esophagus*
**2018**, *15*, 69–74.

The authors state that the scientific conclusions are unaffected. This correction was approved by the Academic Editor. The original publication has also been updated.
